# Benzil/triethylamine: a photo-reducing system for Cu^2+^

**DOI:** 10.1007/s00706-017-2085-7

**Published:** 2018-02-03

**Authors:** Max Schmallegger, Georg Gescheidt

**Affiliations:** Institute of Physical and Theoretical Chemistry, Graz University of Technology, NAWI Graz, Stremayrgasse 9, 8010 Graz, Austria

**Keywords:** Photo-induced electron transfer, Radicals, Metal reduction, Spectroscopy

## Abstract

**Abstract:**

We have investigated the photo-induced reduction of Cu^2+^–Cu^0^ using benzil/triethylamine mixtures. The formation of elemental Cu is indicated by the appearance of its characteristic plasmon absorption peaks at 515 nm and 620 nm. Importantly, the nature of the counterion of the Cu^2+^ salt affects the reduction process. In the presence of Cl^−^, the reduction proceeds faster than with SO_4_^2-^. Photo-induced electron transfer between excited benzil and triethylamine leads to the benzil radical anion, which acts as the reducing agent for Cu^2+^ and generates Cu^0^.

**Graphical abstract:**

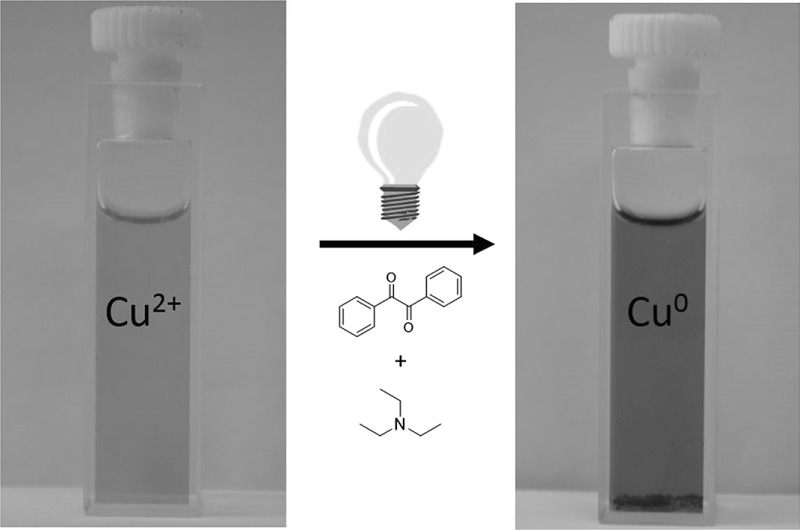

**Electronic supplementary material:**

The online version of this article (10.1007/s00706-017-2085-7) contains supplementary material, which is available to authorized users.

## Introduction

Nanomaterials have experienced a vast growth in interest over the last years. They have been utilized in fields like nonlinear optics and electric conduction [1–3]. Metallic nanoparticles have gained attention because of their remarkable chemical properties, leading to applications, e.g., for molecular imaging [4] or catalysis [5].

A key point for producing nanoparticles is the reduction of metal salts to elemental metals in a controlled way. Chemical, thermal, radiation-chemical, sonochemical, and photochemical methods have been followed in this context [6–9]. However, many of these approaches require expensive reagents, hazardous reaction conditions, and long reaction times combined with difficult isolation procedures [7, 10]. Photochemical methods offer a valuable access to metal reductions allowing temporal and spatial control [11–18]. In such procedures, organic radical anions, produced by photochemical reduction, act as mediators, reducing metal cations to elementary metals [16, 19].

A group of reagents often employed in photo-reductions of metal salts include ketones such as acetophenone [11], acetone [20], and benzophenone [21]. The use of ketones combined with hydrocarbons, alcohols, ethers, and amines has been reported [22, 23]. In such reactions, highly reactive ketyl radicals or ketyl radical anions are formed as intermediates. A substantial requirement for the reducing species is their oxidation potential, since it has to match the potential for the reduction of the metal cation. In terms of metals, the generation of Cu nanoparticles from Cu^2+^ salts has been of prominent interest because of the favorable availability of Cu salts and the activity of Cu as catalyst, in photovoltaics, electronics, and optics [24–27].

The aim of our investigation is to inspect if benzil (1,2-diphenylethane-1,2-dione, **1**) can be utilized for the photo-reduction of Cu^2+^ salts. Benzil is one of the most common (and low cost) diketones and its photochemistry has been well characterized [28, 29]; nevertheless, benzil has yet only seen limited use in photo-induced redox reactions with metal salts.

While the photo-reduction of ketones and diketones in the presence of donor systems, e.g., amines, and the intermediate formation of ketyl radicals are well understood, there are still remaining questions with regard to the mechanism of metal reduction. The mechanisms depicted in Scheme 1 have been suggested for the photo-reduction of metal salts. Here, either ketyl radicals [11, 12, 17, 21] or the ketyl radical anions [30] act as electron donors.

**Figure:**
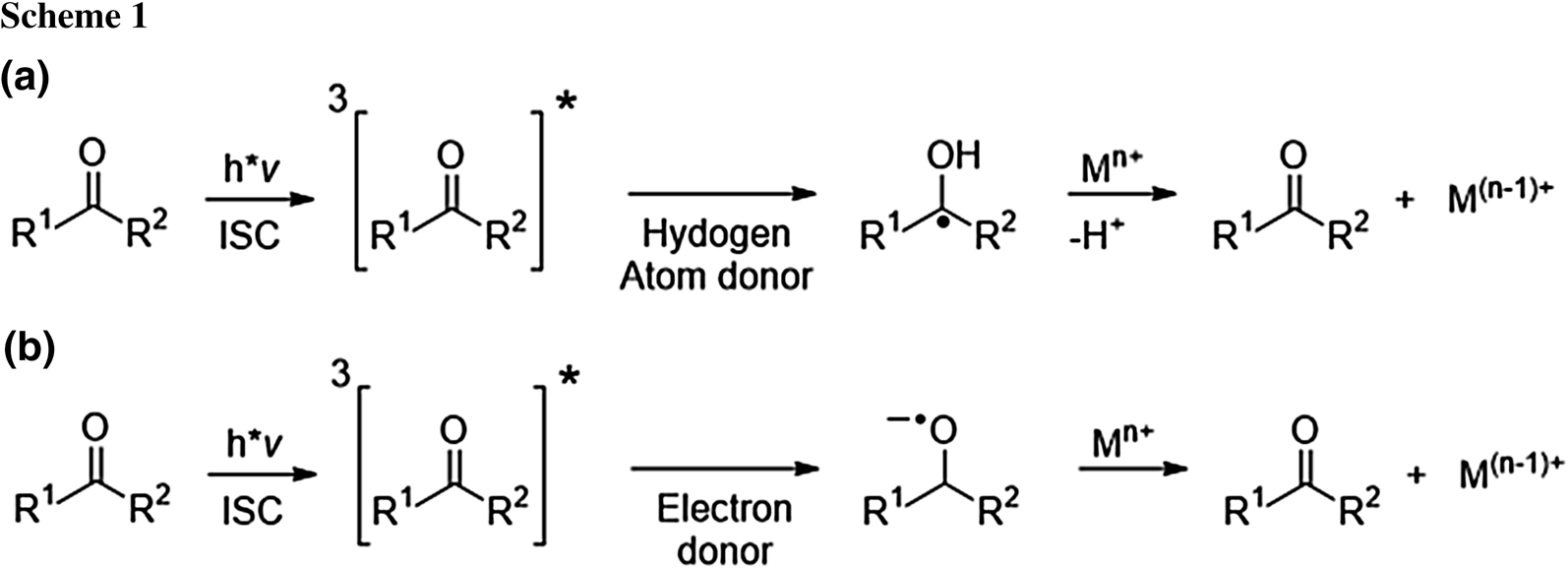

In this publication, we report on the reaction of photo-excited benzil (**1**) and triethylamine (**2**) as a model donor with Cu^2+^ salts. We followed the reactions by steady-state photolysis (SSP), continuous-wave electron paramagnetic resonance (cw-EPR) spectroscopy, and laser-flash photolysis (LFP).

## Results and discussion

### Steady-state photolysis

**Fig. 1:**
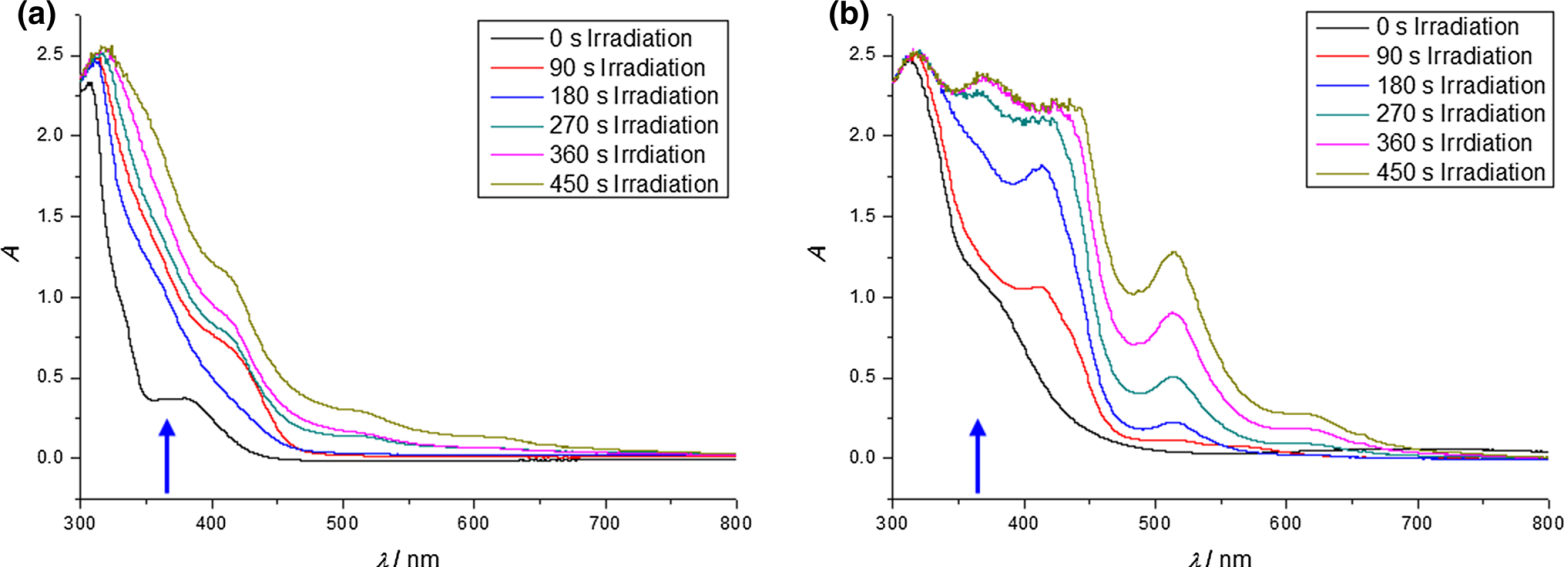
UV–Vis spectrum obtained upon photolysis of **1**/**2** and CuSO_4_ (**a**) or CuCl_2_ (**b**); the arrows represent the irradiation wavelength (365 nm)

We photolyzed (steady state) a solution of **1** and **2** in CH_3_CN containing either CuCl_2_ or CuSO_4_ and observed a new strong band centered at 515 nm and a weaker band at 620 nm (Fig. 1). They can be attributed to characteristic plasmon absorption bands of colloidal Cu [31–35]. It is well established that the plasmon absorption of elemental copper depends on the size of the aggregates formed [34–36]. Therefore, the two absorption bands are in line with an initial formation of small copper aggregates resulting in the band centered at 515 nm, whereas that at 620 nm points to the slower growth of bigger colloids. Control experiments with solutions of the mixtures **1**/**2**, **1**/CuCl_2_ (CuSO_4_), **2**/CuCl_2_ (CuSO_4_), and singly CuCl_2_ or CuSO_4_ (see SI) substantiate these findings. None of these experiments yielded the bands at 515 and 620 nm upon photolysis. The solution **1**/**2** showed absorption spectra indicating the bleaching of the band attributed to parent benzil at 360 nm [37], whereas no spectral changes could be detected for the remaining controls. Accordingly, the copper salts are not decomposed in our irradiation experiments and are inert toward benzil in the absence of the amine and vice versa.

**Fig. 2:**
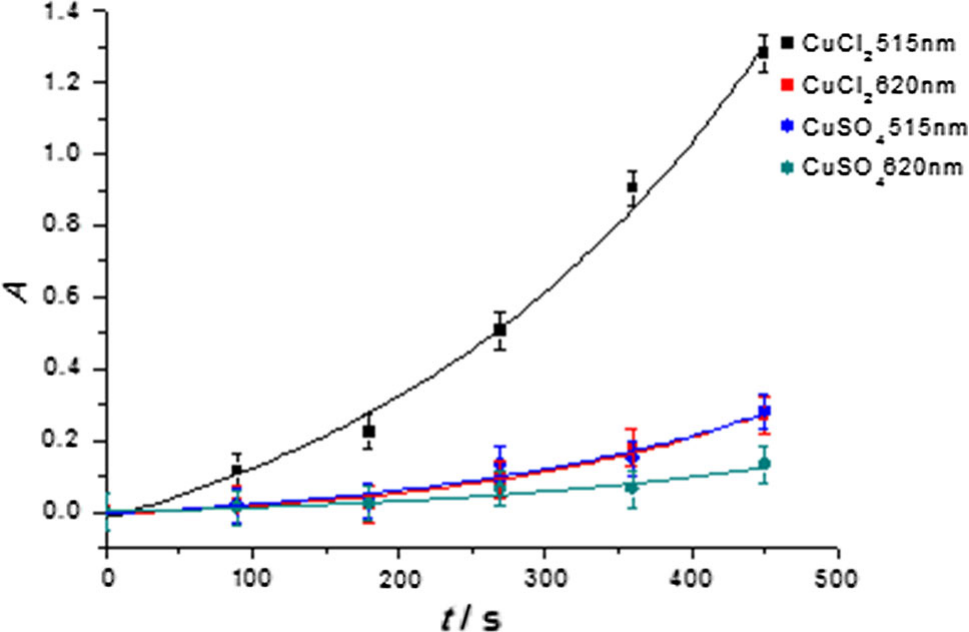
Time trace of the formation of colloidal copper monitored at 515 nm and 620 nm upon photolysis of the **1/2** system and CuCl_2_ or CuSO_4_

Remarkably, the rates for the reduction depend on the counterions of the copper salts (Fig. 2). For CuCl_2_, the bands attributed to the plasmon absorption grow in at a faster rate than for CuSO_4_. This is in line with the results of Pacioni et al. as well as Soares et al., who reported that chloride anions catalyze the disproportionation mediating the conversion of Cu^+^ to Cu^0^ [33, 37]. In addition, electrochemical studies have shown that catalytic amounts of Cl^−^ accelerate the reduction of Cu^2+^ to Cu^+^ [38, 39, 40] (Scheme 2).

**Figure:**
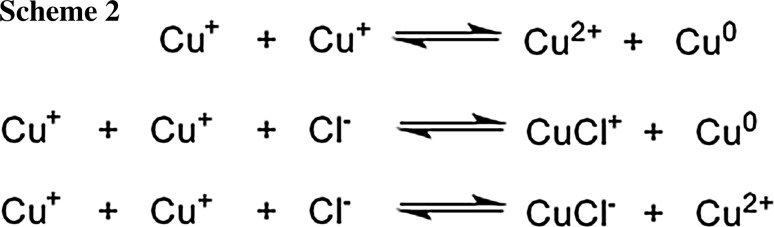

### Continuous-wave electron paramagnetic resonance

**Fig. 3:**
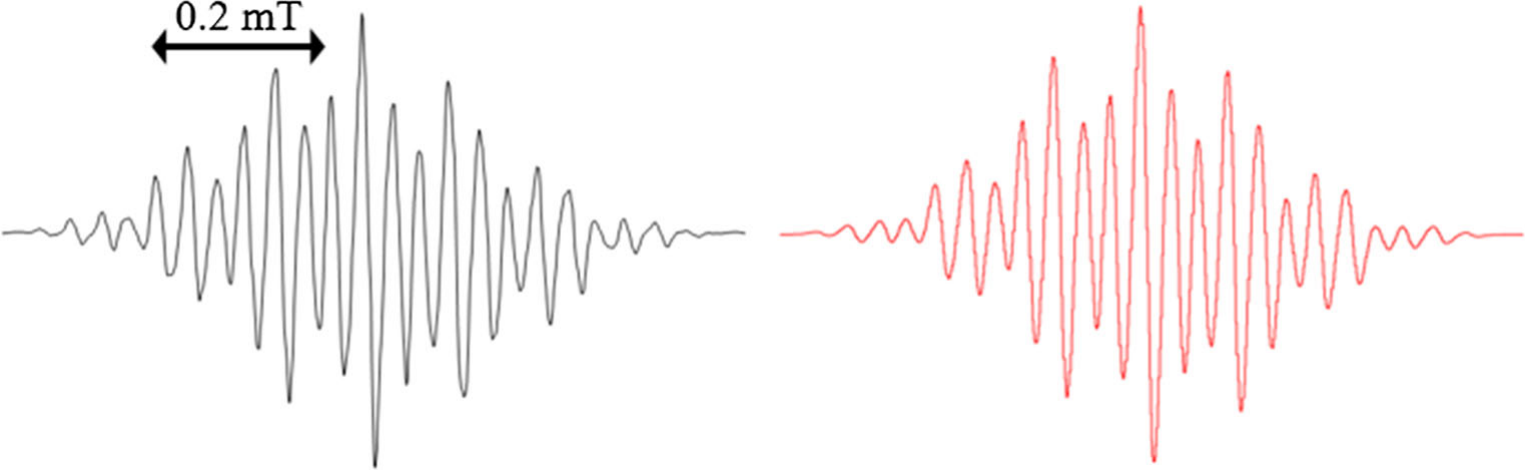
cw-EPR spectrum of **1**^·−^ obtained on the reaction of **1**/**2** in CH_3_CN under continuous irradiation; experimental (left) and simulation (right)

When we observed the solutions of **1** in the presence of **2** in CH_3_CN by cw-EPR under continuous irradiation, we detected the characteristic EPR spectrum attributable to the radical anion of benzil (**1**^·−^). Simulations reveal (Fig. 3, Table 1) an excellent agreement with previously published data [41, 42]. The formation of **1**^·−^ indicates that upon irradiation, **1** undergoes excitation and intersystem crossing to the triplet state 1* [43] and is subsequently reduced by **2**, as indicated in Scheme 3. This is in line with previous studies showing that also for other aromatic ketones such as benzophenone, the corresponding radical anion was observed in photolysis experiments in the presence of alcohols [44] and amines [45].

**Table 1:** Hyperfine coupling constants of **1**^·−^ in CH_3_CN/**2**

Position	hfc/mT	
	CH_3_CN	Literature [42]
*a*_ortho_ (4H)	0.103	0.099
*a*_meta_ (4H)	0.039	0.036
*a*_para_ (2H)	0.108	0.112

**Figure:**
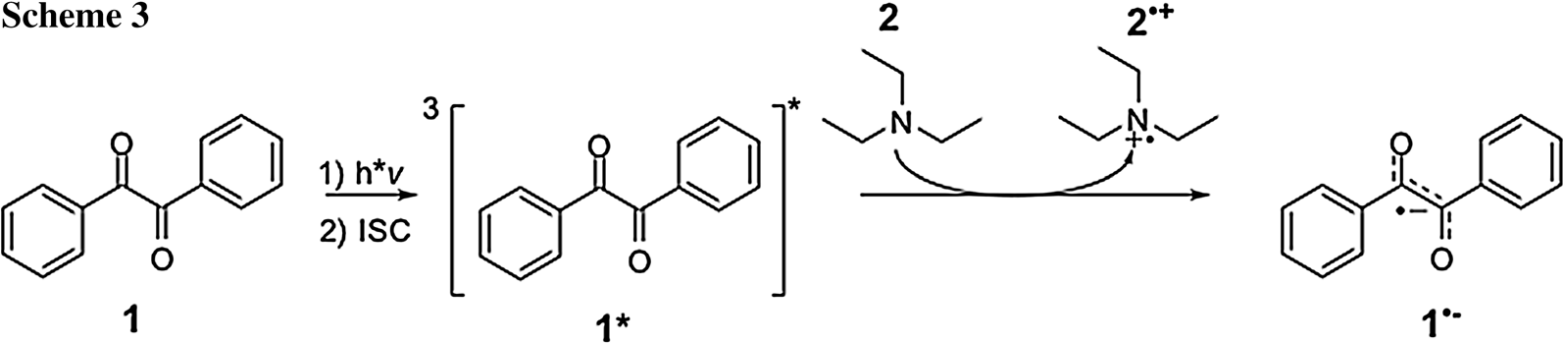

The radical cation of **2**^·+^, formed together with **1**^·−^, is not detected in the EPR spectrum. It is well established that radical cations of amines undergo follow-up reactions, leading to both diamagnetic and paramagnetic species [23, 37, 46, 47] (Scheme 4). Besides parent **2**, the *α*-aminoalkyl radical **4** may serve as an electron-donating species [17, 37, 48]. Therefore, an additional reduction of Cu^2+^ by **4** should be considered when discussing the redox reactions in this system [48]. However, it was shown that **2**^·+^ can undergo rapid follow-up reactions leading to the formation of diamagnetic species [37, 49, 50]. Additionally, product analysis by ^1^H NMR after irradiation (see Supporting Information) reveals formation of *N*,*N*-diethylethenamine (**5**), further rationalizing that the electron transfer from triethylamine-derived radicals only plays a minor role in this system.

**Figure:**
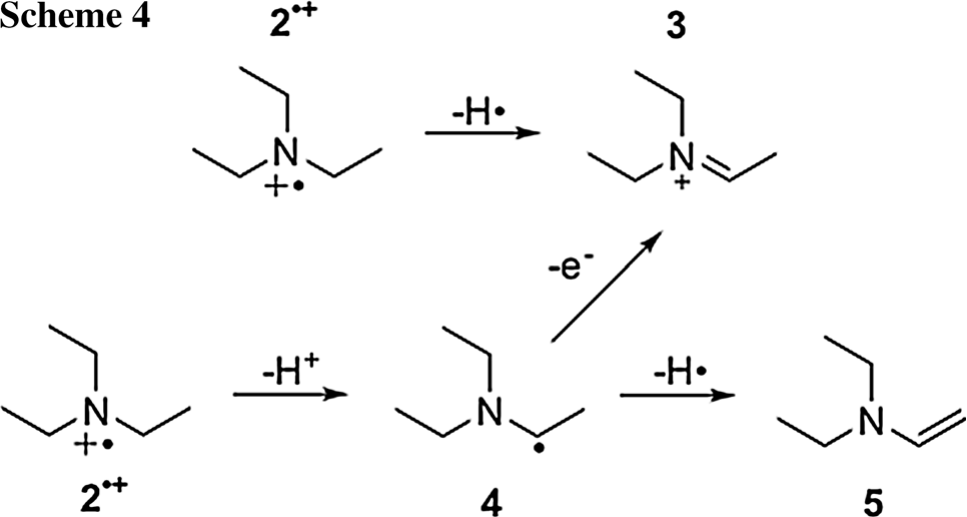

### Laser-flash photolysis

**Fig. 4:**
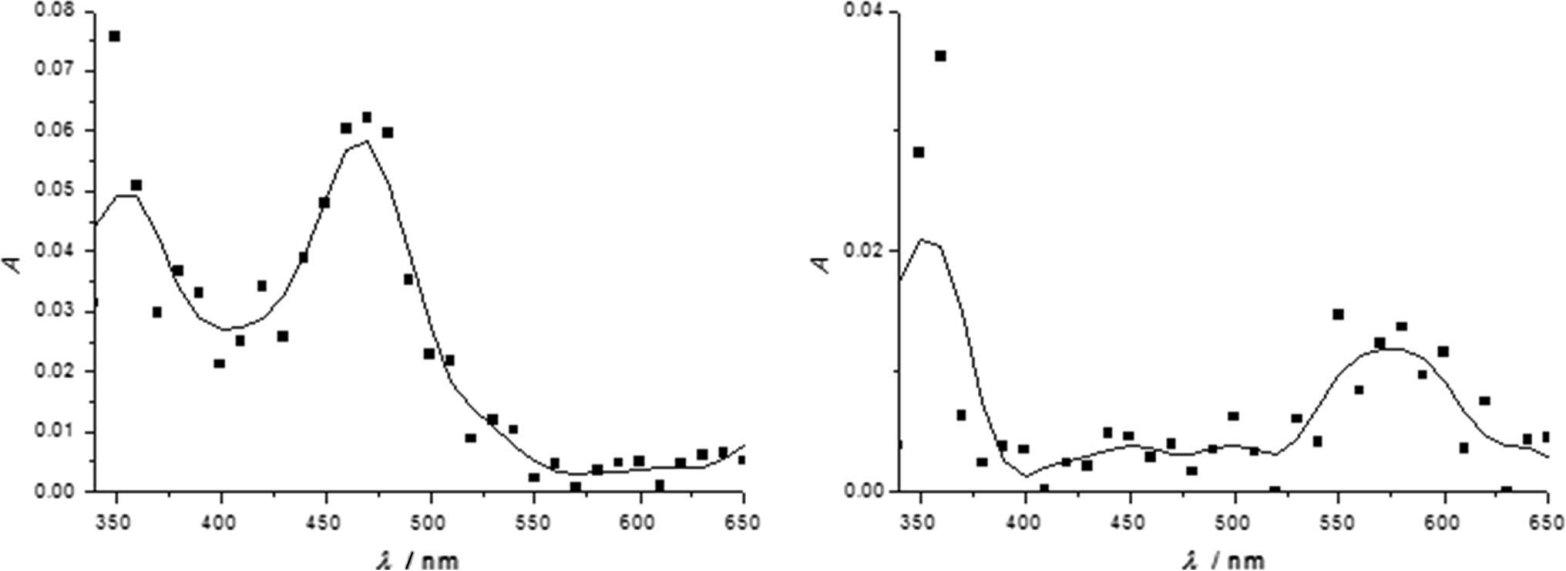
Transient absorption spectra of radicals produced by photolysis of **1** in CH_3_CN (left) and CH_3_CN/**2** 0.2 µs after the laser pulse. See text for the assignment of the absorption bands

To evaluate the role of **2** for the reduction of Cu^2+^ to Cu^0^ at a short (ns) time scale, we carried out laser-flash photolysis (LFP) experiments. Figure 4 shows the transient absorption spectra of **1**/**2** in CH_3_CN and the corresponding reference measurements, in which **2** was omitted. Photolysis of **1** in CH_3_CN yields two distinct peaks at 350 nm and at 480 nm, which are both attributable to 1* [31, 35, 43]. Upon addition of **2** to the solution, significant changes in the spectrum occur: The absorptions centered at 480 and 350 nm disappear, while two new, broad bands centered at 360 and 580 nm appear; they are assigned to **1**^·−^ [51, 52, 53]. This indicates a fast electron transfer reaction of 1* with **2**, leading to the formation of **1**^·−^ and **2**^·+^, respectively [37, 54].

## Conclusions

Our spectroscopic investigation shows that benzil acts as an efficient photo-reducing agent for copper salts in the presence of an amine donor. The efficiency and the rate of the photo-reduction are markedly influenced by the counterions of the copper salts. We have demonstrated the intermediate formation of the benzil radical anion by cw-EPR and LFP. From our experiments, we conclude that **1**^·−^, formed upon photolysis in the presence of **2**, is highly redox active and can reduce Cu^2+^ to Cu^0^. In addition, no indication that the corresponding radical cation of **2** takes part in the reduction of Cu^2+^ to Cu^0^ could be found. This is presumably due to the fast follow-up reaction of **2**^**+**·^, leading to the formation of diamagnetic products that are not redox active.

In future work, this cost-effective and simple approach for the photo-induced reduction of Cu^2+^ to elemental copper could be used for the production of copper nanoparticles by employing different irradiation times as well as characterization methods such as transmission electron microscopy (TEM) to form nanoparticles with defined shape and size [16, 55, 56].

## Experimental

Benzil (**1**, Fluka), triethylamine (**2**, Sigma-Aldrich), copper(II) sulfate (Roth), copper(II) chloride (Riedel-de Haën), and acetonitrilie (Riedel-de Haën) were obtained at the highest purity available and employed as received.

### Steady-state photolysis

UV–Vis spectra were recorded on a fiber optic diode array spectrometer (J&M Analytik AG). Photolysis was conducted using a Hamamatsu Lightingcure LC4 (Hg–Xe lamp, 3500 mW/cm^2^, *λ*_max_ = 365 nm). The concentrations of **1**, CuSO_4_, and CuCl_2_ were 5 mM for all measurements. The concentration of **2** was 100 mM in all measurements. Polyvinylpyrrolidone (10 mg) was added to all samples to help solubilize the copper salts and precipitate the formed Cu^0^.

### EPR spectroscopy

Cw-EPR spectra were recorded on a Bruker X-band spectrometer (EMX, 100 kHz field modulation) at room temperature with 0.025 mT field modulation amplitude. The signals correspond to the steady-state concentration of radicals accumulated in the flow system (0.4 mm quartz flat cell) under continuous irradiation The concentrations of **1** and **2** were 100 and 500 mM, respectively.

### Laser-flash photolysis

LFP experiments were carried out with an LKS80 spectrometer (Applied Photophysics). The excitation of the samples was carried out with the light of a frequency triplet Spitlight Compact 100 (Innolas) Nd:YAG laser at 355 nm (8 ns pulse duration; 10 mJ/pulse energy). The concentration of **1** in solution was adjusted to achieve absorbance of around 0.5 at the excitation wavelength. The concentration of **2** was 100 mM in all measurements.

## ^1^H NMR experiments

^1^H NMR spectra (32 scans) were recorded on a 200 MHz Bruker Avance DPX spectrometer. Chemical shifts (*δ*) are reported in ppm relative to tetramethylsilane (TMS) using the residual undeuterated solvent as an internal reference (acetonitrile-d_3_, *δ*_H_ = 1.94 ppm).

## Electronic supplementary material

Below is the link to the electronic supplementary material.

Supplementary material 1 (DOCX 233 kb)
