# Supplementary material for: Benzil/triethylamine: a photo-reducing system for Cu2+
Source: Monatsh Chem. 2018 Feb 3;149(3):499–504. doi: 10.1007/s00706-017-2085-7 (PMC5859703; doi:10.1007/s00706-017-2085-7)
Supplement: Supplementary file 1 — Supplementary material 1 (DOCX 233 kb) [file 706_2017_2085_MOESM1_ESM.docx]

# Supporting information

# Benzil / Trietylamine: A photo-reducing System for Cu^2+^

M. Schmallegger & G. Gescheidt

Institute of Physical and Theoretical Chemistry,Graz University of Technology, Stremayrgasse 9, 8010 Graz, Austria.

E-Mail: g.gescheidt-demner@tugraz.at


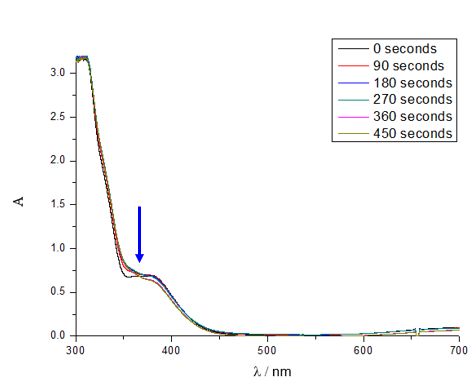


Figure 1. UV-VIS spectrum obtained upon photolysis of 1 in CH_3_CN; the arrow represents the irradiation wavelength (365 nm)


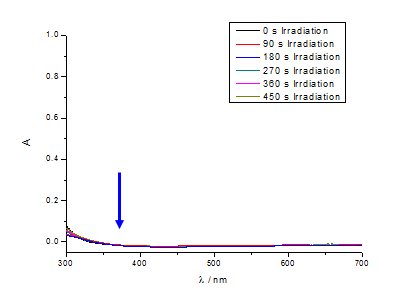


Figure 2. UV-VIS spectrum obtained upon photolysis of CuSO_4_ in CH_3_CN; the arrow represents the irradiation wavelength (365 nm)


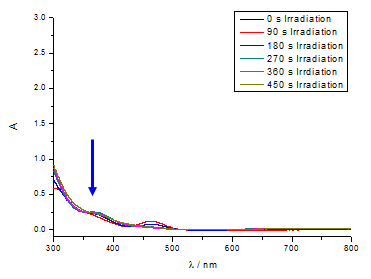


Figure 3. UV-VIS spectrum obtained upon photolysis of CuCl_2_ in CH_3_CN; the arrow represents the irradiation wavelength (365 nm)


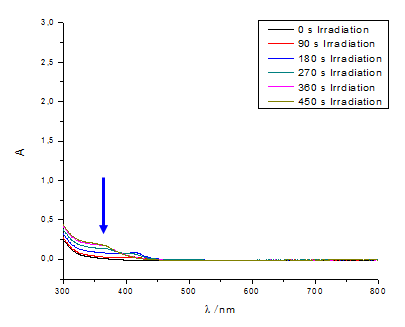


Figure 4. UV-VIS spectrum obtained upon photolysis of CuSO_4_ and 2 in CH_3_CN; the arrow represents the irradiation wavelength (365 nm)


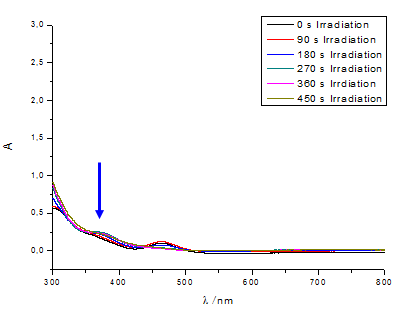


Figure 5. UV-VIS spectrum obtained upon photolysis of CuCl_2_ and 2 in CH_3_CN; the arrow represents the irradiation wavelength (365 nm)


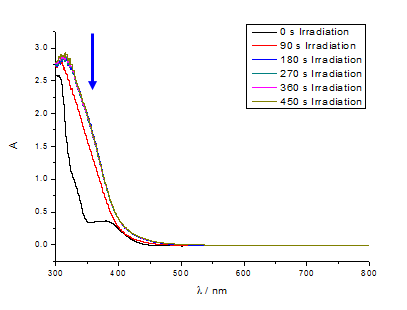


Figure 6. UV-VIS spectrum obtained upon photolysis of CuSO_4_ and 1 in CH_3_CN; the arrow represents the irradiation wavelength (365 nm)


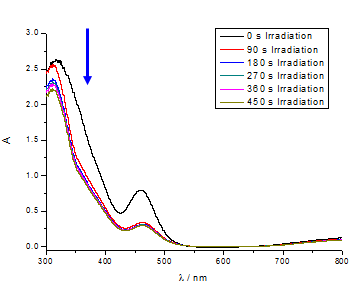


Figure 7. UV-VIS spectrum obtained upon photolysis of CuCl_2_ and 1 in CH_3_CN; the arrow represents the irradiation wavelength (365 nm)


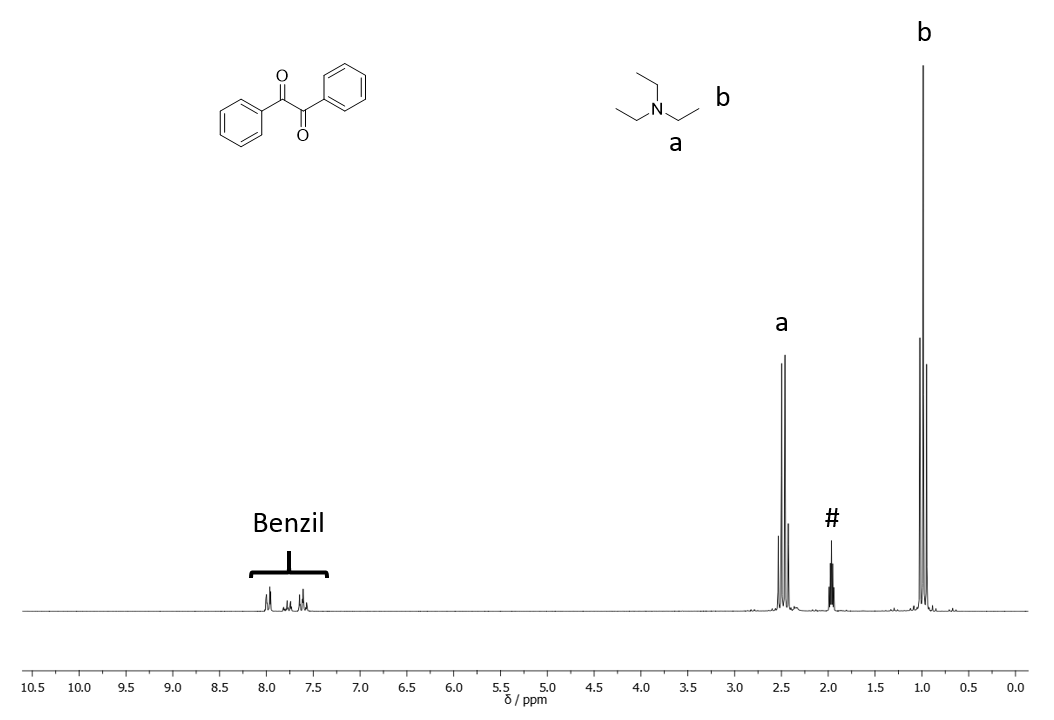


Figure 8. ^1^H NMR of the benzil/TEA system before irradiation, the peak of the solvent is marked by an asterisk


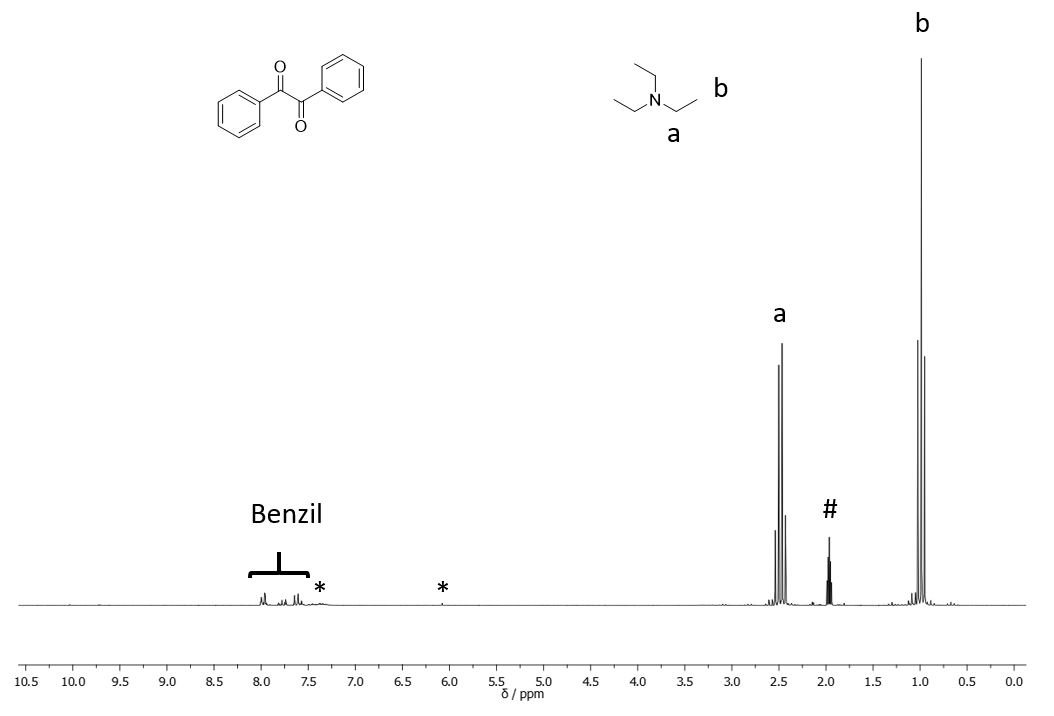


Figure 9. ^1^H NMR of the benzil/TEA system after irradiation, the peak of the solvent is marked by an asterisk; new peaks observable after irradiation are marked by a (*)


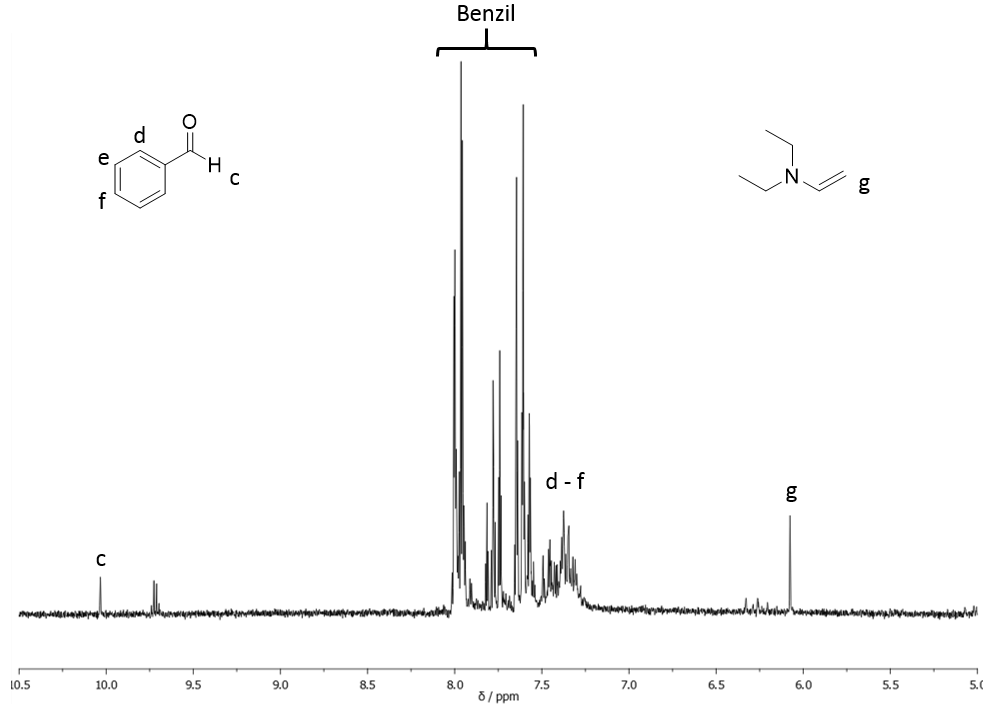


Figure 10. aromatic region of the ^1^H NMR depicted in Figure 4; the formation of benzaldehyde stems from the cleavage of benzil, which is known to occur as a side reaction upon irradiation[1]; the signal at 9.7 ppm is assigned to acetaldehyde formed from the oxidation of triethylamine in the presence of oxygen[2, 3]

References

1. Park JW, Kim EK, Park KK (2002) Bull Korean Chem Soc 23:1229–1234.

2. Cullis CF, Waddingtion DJ (1958). Proc R Soc Lond A Math Phys Sci 244:110–123.

3. Taqui Khan MM, Mirza SA, Bajaj HC (1987). React Kinet Catal Lett 33:67–74.
